# A Comparison of Two Surgical Treatment Methods for Atlantoaxial Instability in Dogs: Finite Element Analysis and a Canine Cadaver Study

**DOI:** 10.3390/ma19020316

**Published:** 2026-01-13

**Authors:** Piotr Trębacz, Mateusz Pawlik, Anna Barteczko, Aleksandra Kurkowska, Agata Piątek, Joanna Bonecka, Jan Frymus, Michał Czopowicz

**Affiliations:** 1 Department of Small Animal Surgery and Anesthesiology, Institute of Veterinary Medicine, Warsaw University of Life Sciences-SGGW, Nowoursynowska 159 C, 02-776 Warsaw, Poland; joanna_bonecka@sggw.edu.pl (J.B.); jan_frymus@sggw.edu.pl (J.F.); 2 Department of Biomaterials and Medical Devices Engineering, Faculty of Biomedical Engineering, Silesian University of Technology, 41-800 Zabrze, Poland; aleksandra.kurkowska@cabiomede.com (A.K.); agata.piatek@polsl.pl (A.P.); 3 CABIOMEDE Ltd., 25-663 Kielce, Poland; 4 Division of Veterinary Epidemiology and Economics, Institute of Veterinary Medicine, Warsaw University of Life Sciences-SGGW, Nowoursynowska 159 C, 02-776 Warsaw, Poland; michal_czopowicz@sggw.edu.pl

**Keywords:** atlantoaxial instability, toy breeds, patient-specific vertebral stabilizer, dorsal fixation, ventral fixation, finite element analysis, biomechanics

## Abstract

Atlantoaxial instability (AAI) in toy- and small-breed dogs remains a significant clinical challenge, as the restricted anatomical space and risk of complications complicate the selection of implants. This study aimed to compare three patient-specific Ti-6Al-4V stabilizers for the C1–C2 region: a clinically used ventral C1–C3 plate, a shortened ventral C1–C2 plate, and a dorsal C1–C2 implant. Computed tomography, segmentation, virtual reduction, CAD/CAM design, and finite element analysis were employed to evaluate the linear-static mechanical behavior of each construct under loading ranging from 5 to 25 N, with a focus on displacements, von Mises stresses, and peri-screw bone strains. Additionally, cadaver procedures were performed in nine small-breed dogs using custom drill guides and additively manufactured implants to evaluate procedural feasibility and implantation time. Finite element models demonstrated that all stabilizers operated within material and biological safety limits. The C1–C3 plate exhibited the highest implant stresses, while the C1–C2 plate demonstrated an intermediate response, and the dorsal implant minimized implant stresses, albeit by increasing bone stresses. Cadaver experiments revealed that dorsal fixation required less implantation time than ventral fixation. Collectively, the findings indicate that all evaluated constructs represent safe stabilization options, and the choice of implant should reflect the preferred load-transfer pathway as well as anatomical or surgical constraints that may limit ventral access.

## 1. Introduction

Atlantoaxial instability (AAI) is a clinically significant disorder that predominantly affects toy-breed and small-breed dogs. It is most commonly associated with congenital abnormalities such as dens aplasia or hypoplasia, ligamentous insufficiency, and other craniovertebral malformations, which together result in pathological motion at the C1–C2 junction. Clinical manifestations range from cervical pain and proprioceptive ataxia to tetraparesis, respiratory compromise, and, in severe cases, sudden death. Disease severity and outcome are influenced by patient age, chronicity of instability, and the presence of concurrent craniovertebral anomalies [1,2,3]. The primary therapeutic objectives in affected dogs are restoration of anatomical alignment, rigid stabilization of the atlantoaxial joint, promotion of arthrodesis, and prevention of vertebral canal compromise. Although conservative management may be considered in selected cases, its limitations—particularly in neurologically affected patients—often necessitate surgical intervention.

Ventral stabilization of the atlantoaxial junction is generally regarded as the reference surgical approach, as it enables direct reduction in the joint, secure fixation within the C1–C2 complex, and reliable preparation of an arthrodesis bed. However, in toy-breed dogs, the ventral surgical corridor is narrow and anatomically demanding, and technical inaccuracies may lead to severe complications involving the airway, esophagus, or adjacent neurovascular structures, thereby increasing perioperative risk in selected patients [1,2]. Cadaveric biomechanical studies comparing atlantoaxial fixation techniques have historically demonstrated high flexural resistance for polymethylmethacrylate (PMMA)-augmented constructs, while also showing that ventral plate-based designs can achieve comparable torsional performance without the disadvantages associated with PMMA polymerization heat and retropharyngeal bulk [3]. These findings support a progressive shift toward plate-based ventral stabilization, particularly in the smallest canine patients, where soft-tissue morbidity is a significant concern [1,3].

Dorsal fixation of the atlantoaxial junction has been described as an alternative surgical option, offering an approach that is anatomically more distant from the airway and esophagus and primarily involves dissection between muscle bellies. Despite these advantages, traditional dorsal techniques have been associated with limited mechanical stability and a risk of iatrogenic injury related to implant placement. Reported complication rates for dorsal atlantoaxial stabilization in dogs range from 20% to 40%, depending on the surgical technique and implant selection [4,5,6]. Consequently, dorsal instrumentation has typically been reserved for specific clinical scenarios in which ventral access is contraindicated or considered excessively risky.

Parallel to developments in surgical techniques, CT-based patient-specific CAD/CAM workflows have undergone substantial advancement. These workflows encompass image segmentation, virtual reduction, screw trajectory planning, drill guide design, and fabrication of custom ventral plates, enabling reproducible translation from preoperative planning to intraoperative execution, often supported by printed bone models [2]. Prospective accuracy studies focused on AAI have demonstrated that patient-specific drill guides significantly reduce the incidence of vertebral canal breach and increase the likelihood of bicortical screw purchase while improving angular fidelity relative to preoperative plans. These findings indicate that planned trajectories can be reliably achieved at the C1–C2 junction [7]. Similar accuracy has been reported for guide-assisted fixation in other regions of the canine cervical spine, even in the presence of narrow pedicles, further supporting the feasibility of precise, guide-enabled fixation in constrained osseous corridors [8]. From a biomechanical modeling perspective, these data support the use of planned screw vectors and lengths as realistic representations of clinical fixation strategies [7,8].

More recently, patient-specific dorsal constructs have been proposed that integrate locked bicortical screw fixation, including transarticular strategies, with plate geometries tailored to posterior cervical anatomy. Finite element analyses (FEAs) of such constructs have reported von Mises stresses below the yield strength of Ti-6Al-4V and sub-millimetric construct displacements under multidirectional loading, suggesting mechanical feasibility within a physiologically relevant range [9]. However, these dorsal computational investigations have generally been conducted independently of ventral stabilization studies, limiting their direct clinical interpretability when both approaches are considered as potential treatment options.

Despite the availability of cadaveric and mechanical evidence supporting ventral plate-based stabilization [1,3], validated patient-specific planning and drill guide accuracy at the atlantoaxial junction [2,7], and promising computational results for dorsal instrumentation [9], a direct and methodologically harmonized comparison of dorsal versus ventral patient-specific constructs in toy-breed dogs is currently lacking. In particular, no study has evaluated both approaches using a unified finite element framework with consistent geometries, material properties, boundary conditions, loading regimens, and outcome measures [1,2,3,7,8,9]. The absence of comparative data limits evidence-based decision-making in very small patient populations, where approach-related risks may significantly influence surgical strategy.

The present study addresses this gap by performing a comparative finite element analysis of patient-specific dorsal and ventral instrumentation for atlantoaxial instability in toy-breed dogs using clinical geometries and a unified computational workflow. Mechanical behavior was evaluated through total construct displacement, equivalent (von Mises) stress within plates, and strain distributions in the implants and peri-screw cortical bone across a standardized loading range of 5–25 N, with particular emphasis on the 25 N condition. By isolating the mechanical consequences of implant topology and anchorage strategy under matched modeling assumptions, this work provides a biomechanical framework for comparing dorsal and ventral stabilization approaches in the context of patient-specific atlantoaxial fixation [1,2,3,9].

## 2. Materials and Methods

### 2.1. Study Design

This was a computational, comparative finite element study evaluating three patient-specific stabilization constructs for canine AAI. Two constructs—the ventral C1–C3 plate and the dorsal C1–C2 stabilizer—were derived directly from real clinical toy-breed cases. To enable a harmonized, vertebra-matched comparison with the dorsal system, a third construct was generated by shortening the clinical ventral plate to a C1–C2-only configuration while maintaining identical screw corridors and plate geometry over the shared segment.

The analysis focused on three co-primary mechanical readouts under controlled loading conditions:

- Total displacement fields (maximum nodal resultant displacement within the modeled construct);
- Equivalent (von Mises) stress in plates;
- Strain in plates and peri-screw bone (reported as implemented in the solver).

All simulations were performed for resultant loads of 5, 10, 15, 20, and 25 N, with 25 N highlighted in the main text.

Patient case chosen for ventral stabilizer: the 5-year-old male chihuahua with tetraparesis and neck pain lasting for 3 months. Patient case chosen for dorsal stabilizer: the 5-month-old male chihuahua with tetraplegia and neck pain lasting for 1 month.

### 2.2. Imaging, Segmentation, and Alignment

A helical CT scan of the head and entire cervical spine was obtained with a slice thickness of 0.625 mm and a bone algorithm, with the patient in a dorsal recumbency. DICOM data were processed in 3D Slicer for semi-automated threshold-based segmentation of the C1–C4 vertebrae. Each vertebra was exported as an independent triangular STL surface. The C1–C2 complex was virtually reduced by a rigid transformation to restore canal continuity and remove residual subluxation prior to implant fitting.

### 2.3. Implant Designs

Three patient-specific plates were modeled on the reduced C1–C2 anatomy, representing both clinical and comparative configurations. Screw vectors were centered within the described safe corridors for the atlas and axis, with explicit avoidance of the vertebral canal and transverse foramina. Locking screws with a core diameter of 1.5 mm were specified for both constructs; the plate thickness was set between 1.5 and 2.0 mm to balance structural rigidity with anatomic clearance. Screws were positioned concentrically within their respective corridors. To ensure numerical robustness in toy-breed geometry and to avoid meshing errors associated with sub-millimetric features, the thread forms were not explicitly modeled in either the screw heads/plate holes or the screw shafts. Instead,

- Locking head–hole engagement was represented by the contact/constraint formulation, a tied/locking interface capturing load transfer without geometric threads (Section 2.9).
- Screw shafts were modeled as smooth cylinders with an effective core (minor) diameter of 1.10 mm (no threads). The bone–thread interaction was likewise represented through the screw–bone contact/constraint definition, which governs axial and shear load transfer in lieu of explicit thread geometry (Section 2.7).
- These abstractions were applied identically to both constructs to preserve the validity of relative comparisons, and they markedly reduced mesh distortion and solver nonlinearity associated with features < 0.4 mm.

Prior AAI mechanical and computational studies informed the selection of corridors and permissible angulations used here.

### 2.4. Ventral C1–C3 Construct (Clinical Configuration)

The first ventral plate spanned C1–C3 (Figure S1, Supplementary Materials), accommodating C1 × 5, C2 × 2, and C3 × 2 screws with coverage adapted to the ventral curvature and anticipated graft bed:

- Ventral arch and body of C1 vertebra (the first five screws)—two screws inserted bicortically to the wings and three monocortically to the ventral arch and body of the C1 vertebra;
- A pair of screws inserted through the C1–C2 intervertebral joints;
- The last pair of screws was inserted transpedicularly in the C3 vertebra.

Ventral plate geometry and planning conventions followed contemporary AAI CAD/CAM practice.

### 2.5. Ventral C1–C2 Construct (Comparative Configuration)

To achieve methodological consistency in finite element comparison, an additional ventral plate was designed to stabilize C1–C2 only, mirroring the vertebral span of the dorsal implant (Figure S2, Supplementary Materials). Screw positioning in C1 replicated the clinical design (five screws placed according to the same corridor geometry), while the C2 segment incorporated a pair of screws inserted through the C1–C2 intervertebral joints and one monocortical screw placed in the caudal part of the body of the axis. This design reduced the fixation span while maintaining plate curvature, thickness, and screw alignment consistent with the original ventral construct, allowing a direct biomechanical comparison between ventral and dorsal two-vertebra constructs under identical boundary conditions.

### 2.6. Dorsal Construct

A dorsal plate covered C1–C2 (Figure S3, Supplementary Materials), integrating C1 × 2 screws + a dorsal hook and C2 × 5 screws to achieve stability across the joint while respecting dorsal safe corridors:

- The hook was hooked onto the dorsal arch of the C1 vertebra;
- The first pair of screws was inserted bicortically through the left and right lateral mass of the C1 vertebrae;
- The second pair of screws was inserted bicortically through the body of the C2 vertebrae;
- The last three screws were inserted bicortically through the spinal process of the C2 vertebrae.

### 2.7. Mesh and Solver Setup

**Figure 1:**
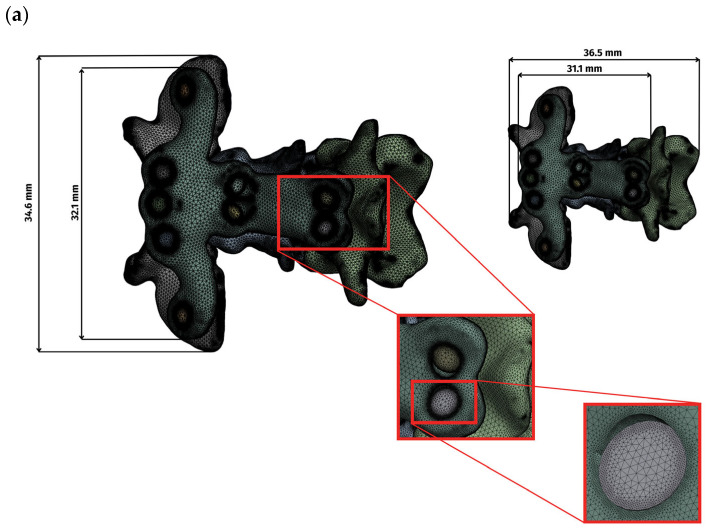

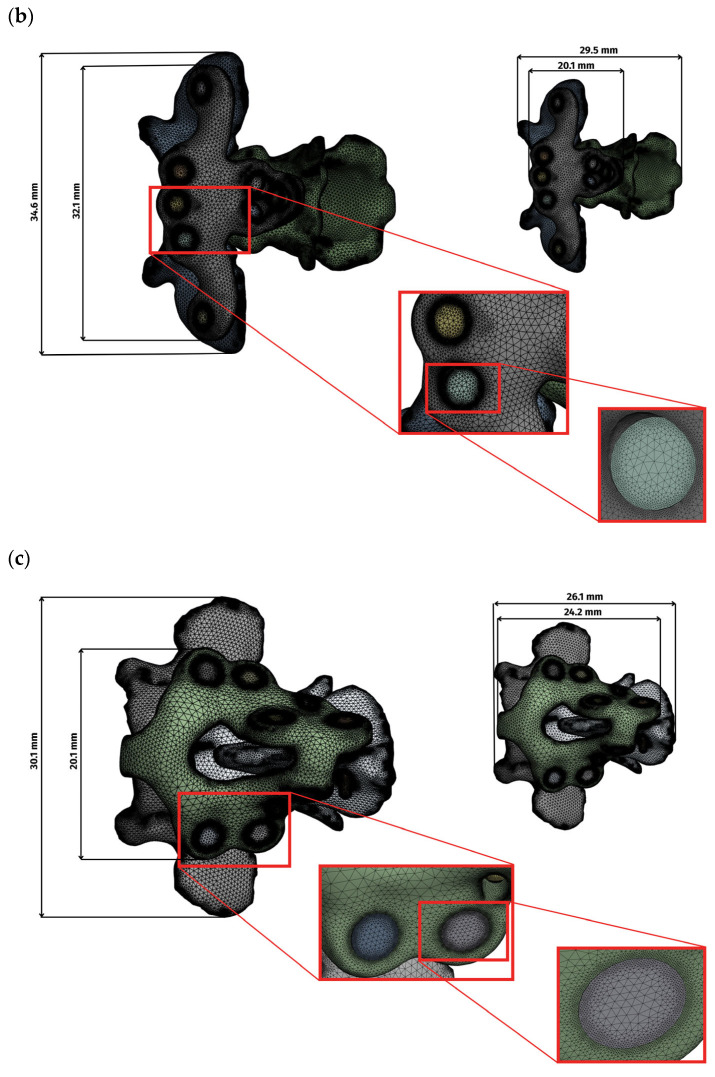
Display of the grid and overall dimensions for each variant: (**a**) ventral construction C1–C3, (**b**) ventral construction C1–C2, (**c**) dorsal construction.

Surface representations of the bone, plates, and screws were converted to volumetric meshes and subjected to standard quality checks before analysis. Preprocessing was performed in Ansys SpaceClaim, and solutions were computed in Ansys Mechanical 2022 R2. A patch-independent mesher was used with four-node linear tetrahedral elements. Element sizing conformed to geometry: locally refined around screw holes, sharp edges, filets, and irregular features, and coarser over smooth, low-curvature regions. For the complete assembly, the total number of mesh elements was as follows: the ventral side (C1–C3) comprised 5,271,336 elements, the ventral side (C1–C2) comprised 4,144,661 elements, and the dorsal side comprised 3,147,846 elements. Mesh sizes for vertebrae, implants, and screws were 0.1–0.5, 0.1–1.0, and 0.1–0.3, respectively, in the dorsal construct, whereas in both ventral constructs (C1–C3 and C1–C2), all sizes were 0.1–0.5 (Figure 1). The overall dimensions of the plate and the complete assembly are presented in Figure 1.

**Figure 2:**
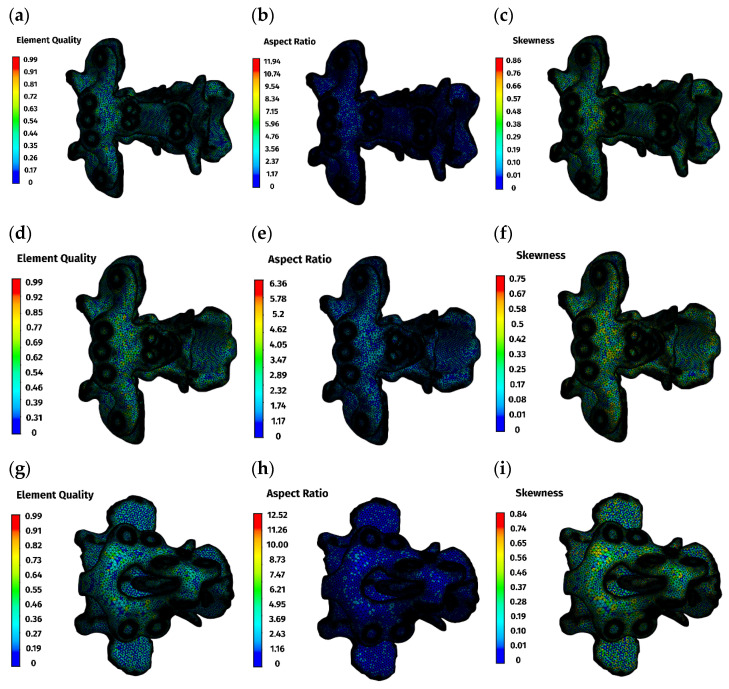
Mesh quality parameters: (**a**–**c**)—ventral construction C1–C3; (**d**–**f**)—ventral construction C1–C2; (**g**–**i**)—dorsal construction.

Mesh quality was evaluated using standard indices (Table 1, Figure 2). Element quality exceeded 0.8 in the majority of elements. Aspect ratio values clustered around 1.9, indicating well-proportioned elements, and skewness peaked at 0.25 and 0.29, indicating low distortion. These values confirm numerical suitability for comparative finite element analysis.

All analyses were performed as linear-static, small-strain simulations with a linear-elastic material framework, using default solver tolerances and iteration controls. Computations were executed on a workstation-class system (Dell Precision 5810, Intel^®^ Xeon^®^ E5-1620 v3, 256 GB RAM, Round Rock, TX, USA). This methodology aligns with prior dorsal FEA studies of atlantoaxial stabilization that emphasize relative construct comparison under controlled boundary conditions.

### 2.8. Material Parameters

Plates and screws were modeled as isotropic Ti-6Al-4V, representative of medical-grade titanium [10]. Linear-elastic behavior was assumed over the applied load range, with literature values assigned for elastic modulus and Poisson’s ratio. No plasticity, damage, or rate effects were included. Cortical bone was represented as isotropic and linear-elastic using literature-appropriate properties for small-breed canine cervical bone [11]. This targets the small-strain regime relevant to the present loading. Material properties were assumed to be temperature-independent (Table 2).

Cancellous bone was not modeled explicitly because, in toy-breed atlantoaxial anatomy, its volume fraction is small relative to the cortical shell. Given the comparative study design and the identical modeling assumptions for the dorsal and ventral constructs, this omission does not bias between-construct inferences.

### 2.9. Contacts and Constraints

Contact idealizations captured locking fixation and plate-bone apposition while maintaining numerical stability. All contacts were specified as surface-to-surface. The bone–bone interface exhibited a frictional behavior (μ = 0.46), approximating physiologic resistance. The bone–plate interface was frictional (μ = 0.3) to represent compressive seating of the plate on the cortex. The bone–screw and plate–screw interfaces were bonded, reflecting locking screw–plate behavior and ensuring full load transfer without slip (Table 3) [12].

Prior dorsal FEA used linear contact formulations with construct-level readouts, supporting the present approach for relative comparison.

### 2.10. Boundary Conditions and Loading

**Figure 3:**
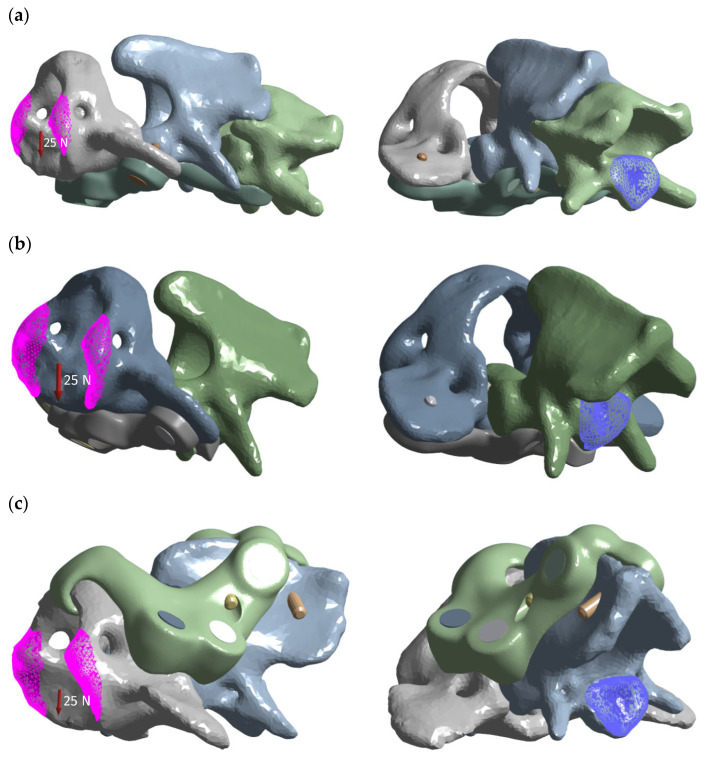
Load distribution graphic for each variant: (**a**) ventral construction C1–C3; (**b**) ventral construction C1–C2; (**c**) dorsal construction.

A common load matrix of 5, 10, 15, 20, and 25 N resultant forces was defined, with 25 N being emphasized in the main text. In all cases, force was applied to C1 at a defined cranial surface region, while the fixed support was placed on the caudal surface of C2 or C3 (Figure 3).

This schema is compatible with boundary-condition variants reported in dorsal AAI simulations while standardizing constraints per construct for comparability.

### 2.11. Outputs and Post-Processing

Maximum resultant displacement (mm) was extracted separately for each component (plate, bone) by isolating the element in the Ansys Mechanical post-processor and applying the “adjust to visible” function. Peak values were averaged across several adjacent elements to minimize sensitivity to local mesh irregularities. This approach ensured that reported maxima reflected component-level behavior rather than mesh-dependent local artifacts.

Von Mises stress (MPa) was computed for plates and vertebrae. Equivalent strain fields were calculated in plates and peri-screw cortical bone to visualize potential regions of risk for microfracture or loosening. Displacement and stress/strain heatmaps were generated for a load of 25 N. Reporting these readouts aligns with contemporary AAI mechanics and computational analyses.

### 2.12. Cadaver Study

In a cadaver study, the time of the surgical procedure was measured from the moment the skin was incised to the moment the stabilizer was screwed in. The study included the small-breed and toy-breed dogs, with no pathologies of the C1–C2 junction. Dogs were euthanized for reasons unrelated to this study. Immediately after euthanasia, the CT scans of the C1–C2 junction were made. Then the cadavers were frozen at −20 °C. For each cadaver, sets of drill guides and stabilizers for the ventral and dorsal regions of the C1–C2 junction were printed from resin using digital printing techniques based on computed tomography images. A day before the surgical procedure, the cadavers were removed from the freezer and allowed to thaw at room temperature. According to Polish legislation, ethics approval was not required for this study (Act of the Polish Parliament of 15 January 2015 on the Protection of Animals Used for Scientific or Educational Purposes, Journal of Laws 2015, item 266) [13].

**Figure 4:**
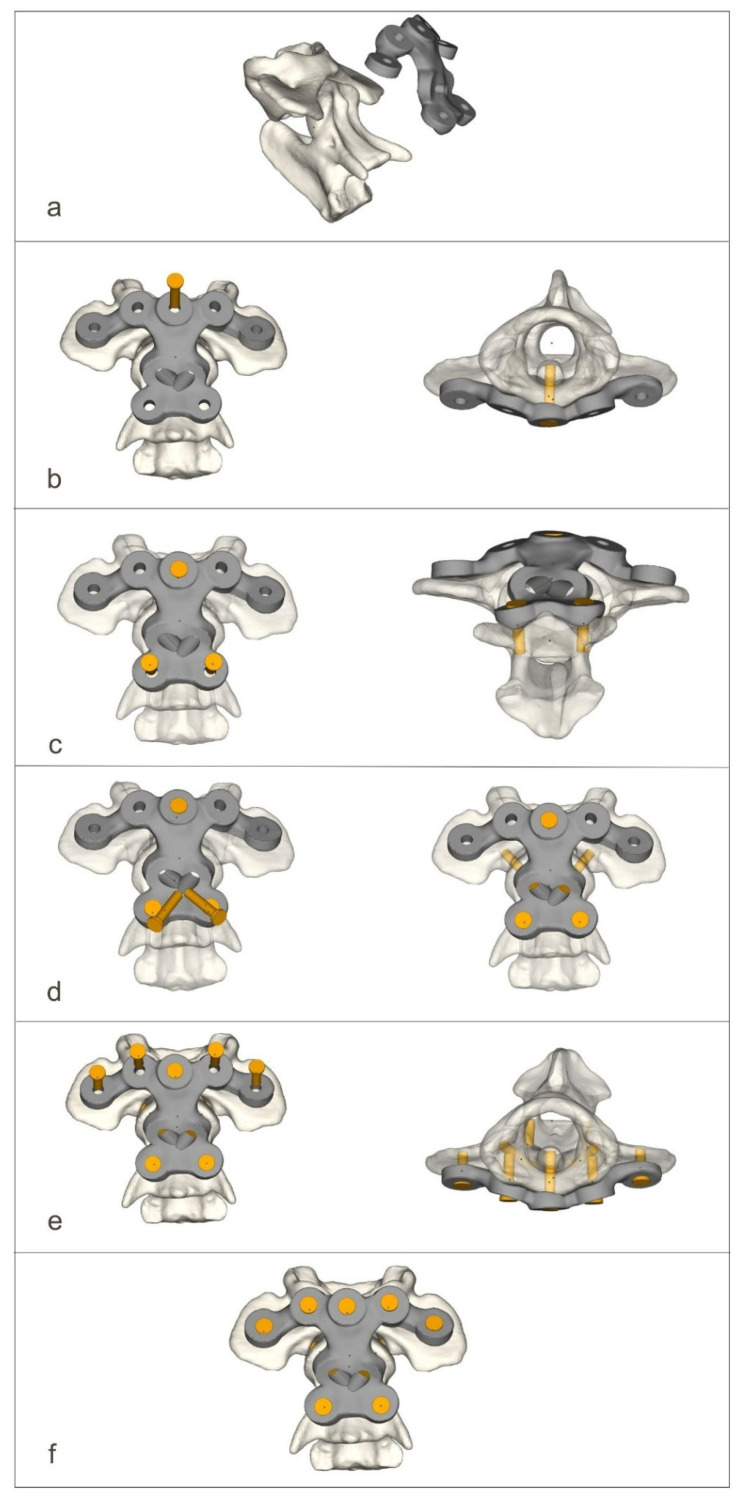
The process of screwing on the ventral stabilizer (**a**–**f**).

The ventral stabilizers were inserted first. For this approach, the dogs were positioned in dorsal recumbency with their necks extended. The median approach to the C1–C3 region was then performed [14]. After exposition, holes were drilled ventrodorsally using the drill guide. After inserting the stabilizer, the screws were then screwed into the order shown in the figure (Figure 4).

**Figure 5:**
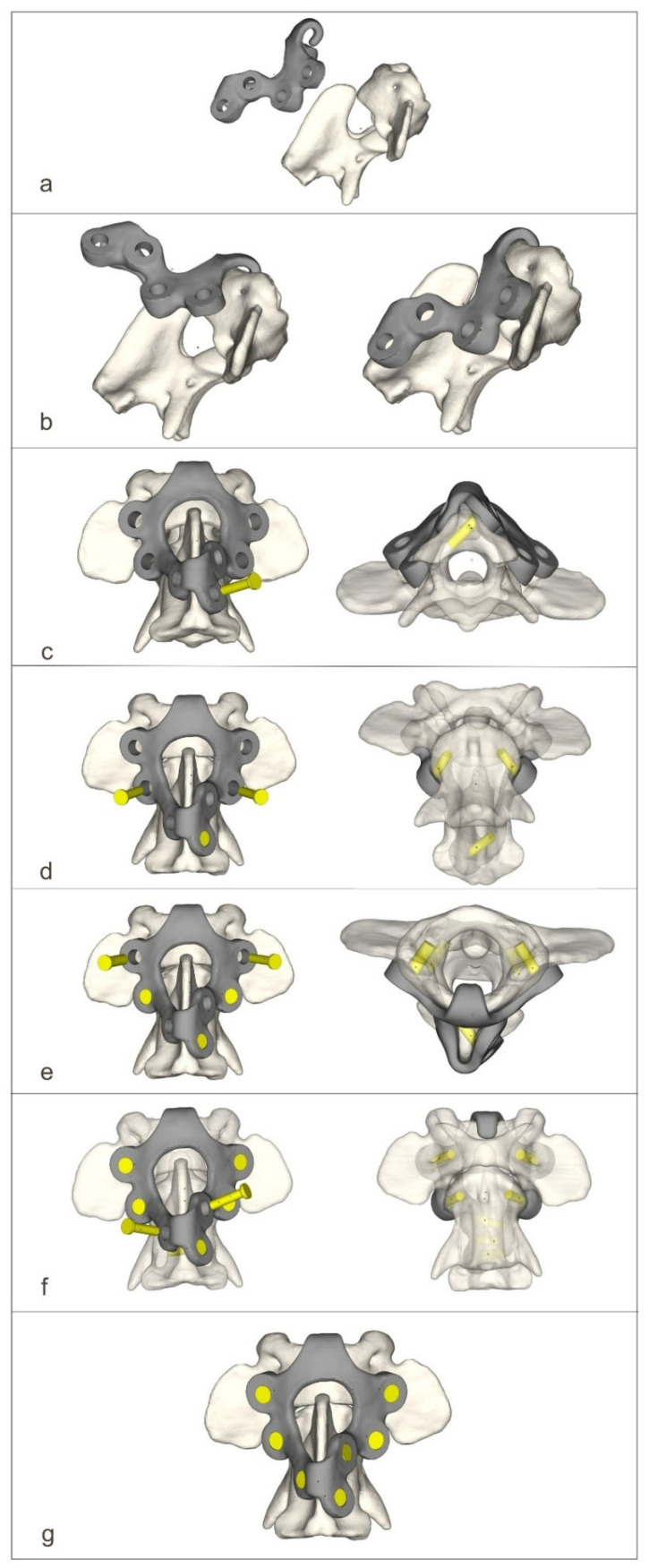
The process of screwing on the dorsal stabilizer (**a**–**g**).

For the dorsal approach, the dogs were positioned in ventral recumbency with their necks flexed. The median approach to the C1–C2 junction with the wide exposure of the dorsal arch of the C1 and spinous process of the C2 vertebrae was executed [6]. After exposition, holes were drilled dorsoventrally using the drill guide. After inserting the stabilizer, the screws were then screwed into the order shown in the figure (Figure 5).

### 2.13. Statistical Methods (Cadaver Study)

Numerical variables (body weight, time needed for stabilization at 2 approaches) were examined for normality of distribution using normal probability Q-Q plots and the Shapiro–Wilk test. As they proved to be normally distributed, they were summarized as the arithmetic mean, standard deviation (SD), and range. Times needed for stabilization were compared between the two approaches using the paired Student’s *t* test and between crossbreeds and pedigree dogs using the unpaired Student’s *t* test. The correlation between body weight and the time needed for stabilization at two approaches was examined using the Pearson linear correlation coefficient (r), and the 95% confidence interval (CI 95%) was calculated according to Altman et al. [15]. All tests were two-tailed, and a significance level (α) was set at 0.05. The analysis was performed in TIBCO Statistica 13.3 (TIBCO Software Inc., Palo Alto, CA, USA).

## 3. Results

### 3.1. Finite Element Analysis

The finite element analysis provided a detailed evaluation of stress, strain, and displacement behavior across the three patient-specific stabilization constructs: the clinical ventral C1–C3 plate, the comparative ventral C1–C2 plate, and the comparative dorsal C1–C2 stabilizer. All constructs demonstrated a linear mechanical response within the applied loading range of 5–25 N, which confirmed the elastic behavior of both bone and implant materials, as well as the numerical robustness of the simulations—Table 4 (Figure S4, Supplementary Materials).

**Figure 6:**
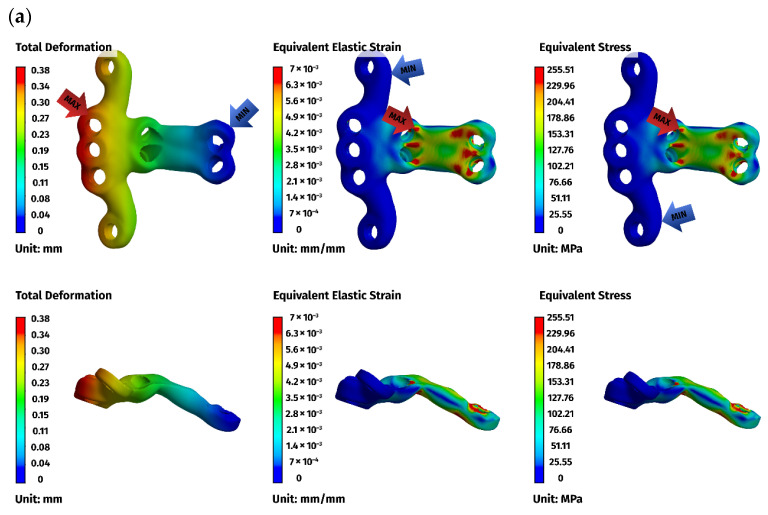

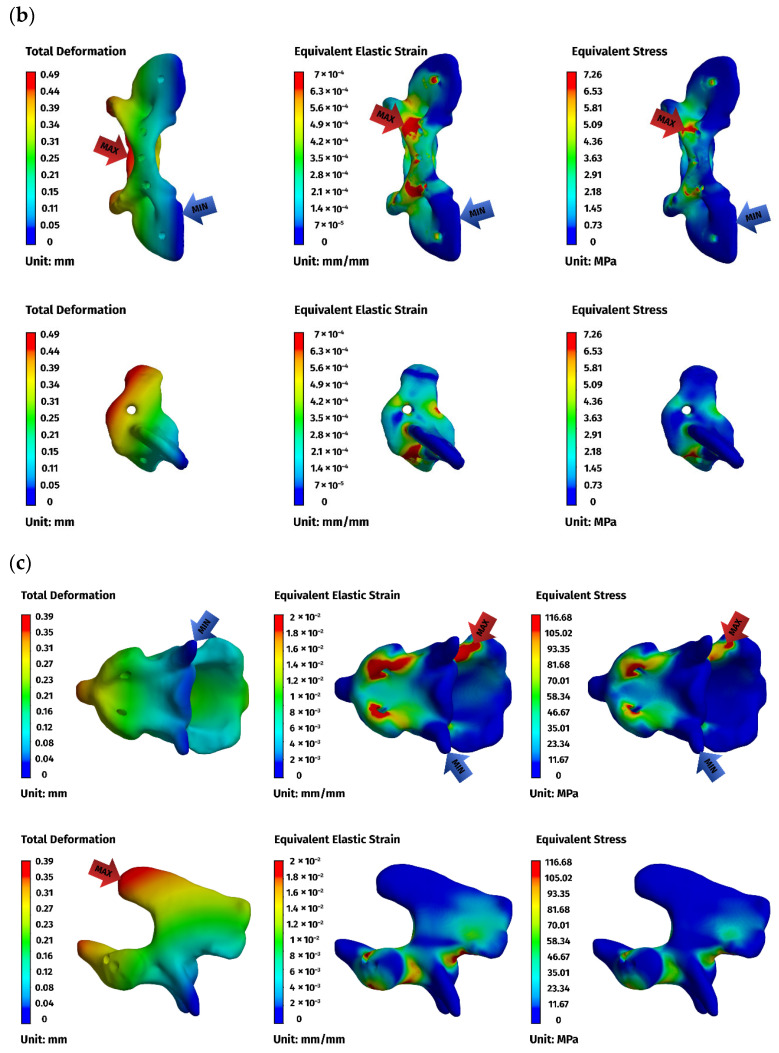

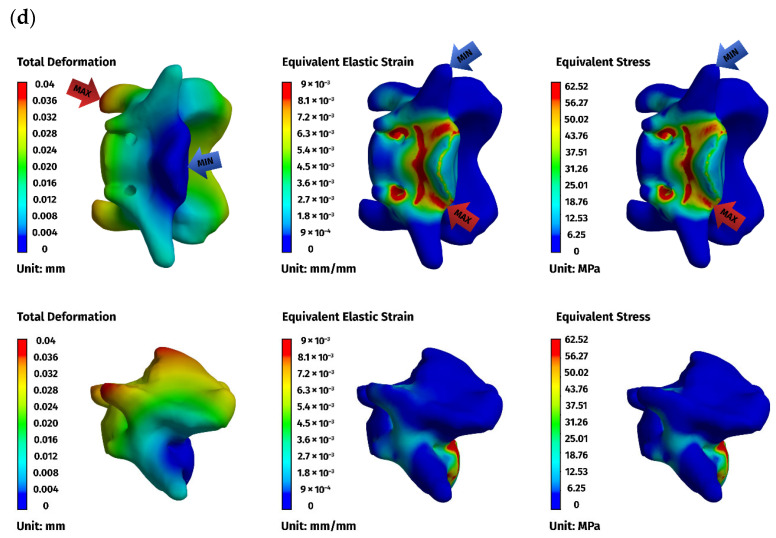
Finite element method (FEM) results for the ventral stabilizer C1–C3: (**a**) plate, (**b**) C1, (**c**) C2, (**d**) C3. The red arrows indicate the regions of maximum deformation, strain, and stress, while the blue arrows mark the areas where these values reach their minimum.

Across all models, displacements increased in proportion to the magnitude of the load. At lower loads (5 N), displacements were minimal, typically ranging from 0.01 to 0.08 mm, while at the highest load of 25 N, values ranged from 0.02 to 0.49 mm. The largest deformations occurred in the ventral C1–C3 construct, particularly at the level of C1, reflecting the longer lever arm associated with a three-vertebra fixation. In contrast, both C1–C2 constructs displayed considerably smaller displacements and a more compact deformation pattern, consistent with their shorter fixation span (Figure 6a).

Implant stresses varied substantially between constructs. The ventral C1–C3 stabilizer consistently exhibited the highest von Mises stresses, increasing from 58.83 MPa at 5 N to 255.51 MPa at 25 N, indicating that this implant configuration absorbed a significant proportion of the applied load. The ventral C1–C2 plate demonstrated intermediate implant stresses, rising from 11.44 MPa to 62.21 MPa across the load range. The dorsal C1–C2 stabilizer showed the lowest implant stresses of all three designs, with values ranging only from 4.7 MPa to 24.29 MPa. These patterns indicate that shortening the ventral stabilizer reduced its mechanical demand, while the dorsal design transferred most of the load away from the implant and into the supporting bone.

Vertebral stresses complemented the implant findings. In the dorsal construct, stresses in C1 and C2 were consistently the highest, peaking at 38.32 MPa in C1 and 26.78 MPa in C2 under the 25 N load. The ventral C1–C2 construct produced moderate bone stresses, whereas the ventral C1–C3 construct generated the lowest stresses in both C1 and C2 due to redistribution of loads towards the distal C3 segment, which itself experienced stresses up to 62.52 MPa. Despite these differences, all vertebral stress values remained below the expected cortical yield threshold for toy-breed cervical bone, confirming that each construct operated within safe biomechanical limits under the examined loading conditions.

**Figure 7:**
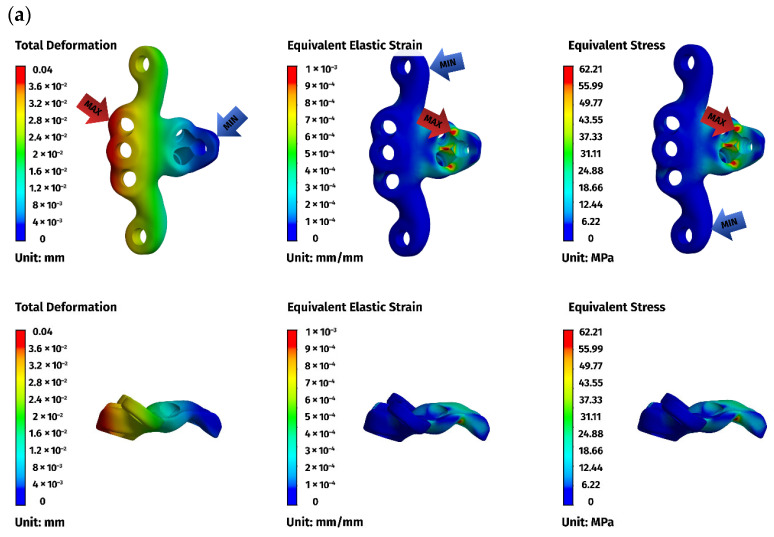

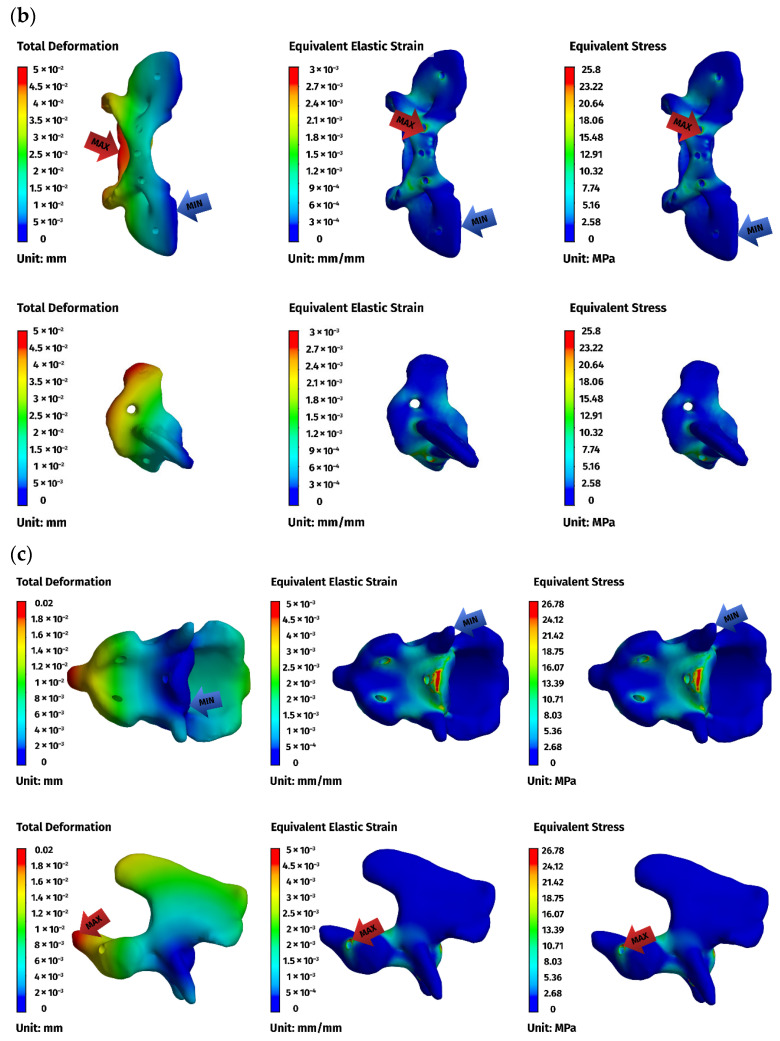
FEM results for the ventral construction C1–C2: (**a**) plate, (**b**) C1, (**c**) C2. The red arrows indicate the regions of maximum deformation, strain, and stress, while the blue arrows mark the areas where these values reach their minimum.

**Figure 8:**
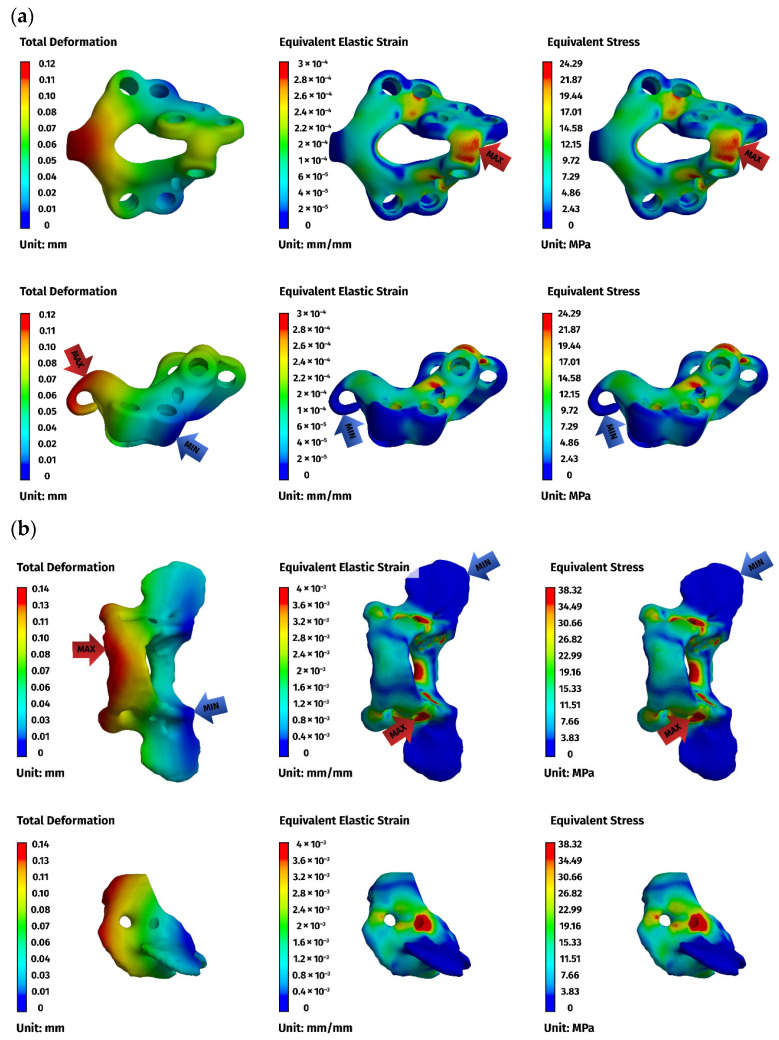

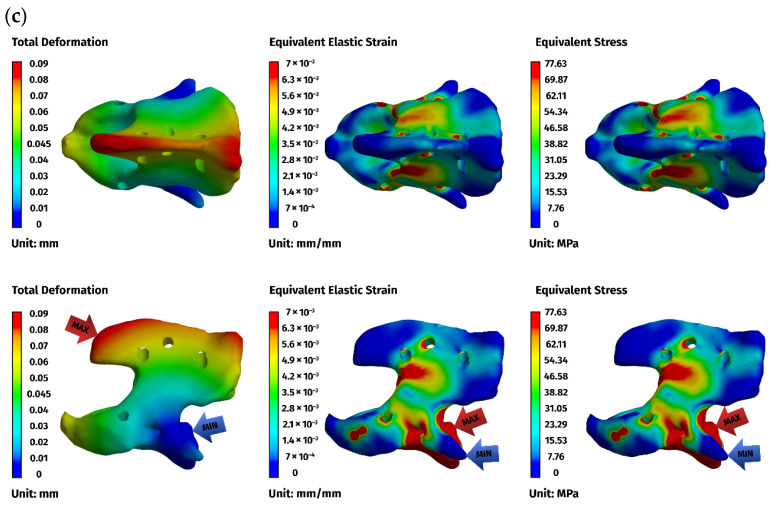
FEM results for the dorsal construction: (**a**) plate, (**b**) C1, (**c**) C2. The red arrows indicate the regions of maximum deformation, strain, and stress, while the blue arrows mark the areas where these values reach their minimum.

Overall, the analysis revealed a clear mechanistic distinction between the three stabilization strategies. The ventral C1–C3 construct carried higher implant stresses but reduced vertebral loading by distributing forces across three segments. The ventral C1–C2 construct produced a mixed load-sharing pattern, with both the plate and vertebrae bearing moderate stress levels. The dorsal C1–C2 stabilizer resulted in the lowest implant stresses but the highest stresses within C1 and C2, reflecting a bone-centric load-transfer pathway. Despite these differences, all constructs provided sufficient stiffness and maintained mechanical integrity across the physiologically relevant loading regime. The detailed results for the 25 N load are presented below (Figure 6, Figure 7 and Figure 8).

### 3.2. Cadaver Study

The study included cadavers of 9 dogs, 8 males, and 1 female. There were 4 crossbreed dogs, 2 Yorkshire terriers, a Cairn terrier, a West Highland white terrier, and a miniature poodle. Body weight ranged from 3 to 12 kg (mean ± SD: 6.9 ± 3.0 kg). Body weight did not differ significantly between 4 crossbreeds (8.4 ± 3.0 kg) and 5 pedigree dogs (5.7 ± 2.8 kg) (*p* = 0.203).

Time needed to stabilize C1–C2 ranged from 18.5 to 24.2 min (mean ± SD: 21.2 ± 1.8 min) for ventral approach and from 11.3 to 14.2 min (mean ± SD: 12.9 ± 1.0 min) for dorsal approach. The mean time (±SD) required for the dorsal approach was significantly shorter than that for the ventral approach by 8.3 ± 1.4 min (CI 95%: 7.2 to 9.4 min; *p* < 0.001). There was no significant difference in time needed to stabilize C1–C2 between crossbreed dogs and pedigree dogs, either in ventral approach (21.3 ± 2.0 min vs. 21.0 ± 1.8 min; *p* = 0.849) or dorsal approach (13.0 ± 0.9 min vs. 12.8 ± 1.1 min; *p* = 0.754).

In both the ventral and dorsal approach, the tendency for positive correlation between the time needed to stabilize C1–C2 and the body weight of operated dogs was observed (r = 0.59, CI 95%: −0.13 to 0.90 and r = 0.41, CI 95%: −0.35 to 0.84, respectively); however, none was statistically significant (*p* = 0.097 and *p* = 0.276, respectively), likely due to a small number of dogs included in the study.

## 4. Discussion

### 4.1. Finite Element Analysis

AAI is a state of anatomical malarticulation between the first and second cervical vertebrae that can result in atlantoaxial subluxation and compression of the spinal cord. In addition, there are other less well-described abnormalities of the cervical spine, thought to be associated with AAI, that have been clinically observed, such as C2–C3 instability.

In this study, we employed a computational technique and instrumentation on cadavers to compare the dorsal and ventral stabilization of the atlantoaxial region in dogs. FEA was used to simulate the distribution of stresses and displacements of the implant after applying force to assess its safety and mechanical feasibility. However, cyclic loading and fatigue characteristics were not taken into account. The FEA demonstrates that dorsal and ventral instrumentation at the C1–C2 junction exhibit comparable mechanical performance in small dogs. The ventral approach is more popular as it facilitates odontoidectomy if required and allows for permanent fusion of the atlantoaxial joint (arthrodesis). However, the technical constraints associated with ventral instrumentation mainly concern the limited bone available for implant purchase and the proximity of critical structures, which makes this procedure challenging. Accurate reduction and implant placement are essential for a satisfactory outcome [16]. Despite the advantages of arthrodesis, it is unclear whether complete atlantoaxial arthrodesis is necessary for atlantoaxial stabilization [4,16]. Additionally, Tabanez et al. [6] performed dorsal instrumentation with screw insertion and bone cement. This achieved a comparable success rate to ventral access techniques while promoting stiffness compatible with bone remodeling and vertebral fusion. In our study, the ventral construct was the only one that permitted transarticular screw insertion to promote arthrodesis. Dorsal instrumentation did not facilitate transarticular screw insertion. Nevertheless, the results of the FEA of both stabilizers were comparable.

With the introduction of a third construct—a shortened ventral C1–C2 plate designed to match the dorsal fixation span—the comparative analysis allowed a more detailed evaluation of how fixation length influences mechanical behavior. The three models demonstrated linear behavior within the applied load range, but differed markedly in their internal load-transfer mechanisms. The ventral C1–C3 construct concentrated the mechanical load primarily within the implant, producing the highest plate stresses, whereas stresses in C1 and C2 remained comparatively low due to the redistribution of forces along the extended fixation that engaged three vertebrae. In contrast, the dorsal C1–C2 construct exhibited the lowest stresses within the implant but the highest stresses in the vertebrae, reflecting a bone-centric load pathway associated with its shorter anchorage span and more limited bone–implant interface.

The intermediate ventral C1–C2 construct demonstrated a transitional pattern between these two extremes. By limiting fixation to C1 and C2 while maintaining the ventral implant geometry, this configuration produced lower implant stresses than the long ventral plate, yet higher vertebral stresses (C1) than the extended C1–C3 construct. These findings confirm that fixation span is a major determinant of stress distribution in atlantoaxial stabilization: engaging an additional vertebra reduces the mechanical demand on the C1–C2 segment, whereas restricting fixation to two vertebrae concentrates the load.

A localized non-proportional strain response was observed in one stabilizer configuration at intermediate load levels, manifesting as an apparent outlier in the strain–load relationship. This phenomenon is transitional in nature and occurs only within a limited load range, where a temporary change in the local deformation mechanism and strain accumulation arises due to the specific geometry and boundary conditions of the model. At this stage, a momentary deviation from linear strain behavior can be observed; however, once higher load levels are reached (25 N), the deformation mechanism stabilizes and returns to a linear response. Importantly, even the maximum strain values remain relatively low and well within the elastic range of the material. Consequently, this localized non-proportional behavior does not affect the strength analysis, mechanical safety assessment, or the final conclusions of the study.

From a mechanical standpoint, all three constructs remained within safe stress limits (the yield strength of Ti-6Al-4V is approximately 980 MPa [17]) and provided similar overall stiffness, indicating adequate stabilization potential for small-breed atlantoaxial junctions under physiologic load conditions.

Based on additional literature and fundamental mechanical considerations, it can be anticipated that larger animal models, e.g., larger and heavier dogs, would be primarily subjected to higher bending and torsional moments at the C1–C2 segment, resulting from increased head mass and longer lever arms. Consequently, if implant geometry and screw dimensions were kept unchanged, higher peak stresses in both the implant and the surrounding bone, as well as larger displacements, would be expected, approximately scaling with the applied load within a linear-elastic framework, as observed in the present 5–25 N simulations. However, larger animals typically provide greater bone cross-sectional areas and wider safe corridors, allowing the use of larger-diameter screws and/or thicker plates, which substantially increase construct stiffness and load-bearing capacity. Supporting evidence is provided by Cabreira et al. [9], who developed a patient-specific dorsal implant using ∅ 1.7 mm locking screws and reported a maximum implant stress of approximately 425 MPa (well below the material yield strength) together with small displacements under multidirectional loading (flexion, extension, lateral bending, and torsion). These findings suggest that scaling the construct to larger animal models does not necessarily compromise mechanical safety margins, provided that implant geometry and fixation parameters are appropriately adapted to the increased loading conditions.

Nevertheless, the differences in fixation span and load-sharing behavior have important clinical implications. The ventral C1–C3 construct, by engaging a third cervical vertebra, may mitigate the risk of localized cortical overload and long-term bone resorption at the C1–C2 level. The ventral C1–C2 construct offers an acceptable compromise when extending fixation to C3 is not desirable or anatomically feasible. The dorsal construct, while mechanically feasible and associated with less invasive surgical access, relies more heavily on the bone itself and may therefore be influenced by variations in cortical thickness or developmental anomalies, such as congenital hypoplasia of the atlas.

The dorsal fixation demonstrates mechanical feasibility, but it results in higher osseous loading due to its shorter anchorage span. In contrast, the ventral configuration provides a more distributed and balanced stress environment by engaging three vertebrae. Both approaches remain within mechanical safety margins, and their selection should take into account anatomical constraints, bone quality, and surgical accessibility.

### 4.2. Three-Dimensional Printing of Stabilizers

For the manufacturing of the stabilizers, reverse engineering and computer-aided design and manufacturing (CAD/CAM) techniques were employed. The traditional process of designing a new engineering structure involves a specific sequence of steps. First, the problem must be defined, and the boundary conditions must be specified. Next, a concept is developed, and preliminary designs are produced. The best solution is then selected and a preliminary design developed. Finally, the concept is verified computationally, and any necessary corrections are made. The final design and technical documentation are then prepared. Another method of creating objects is reverse engineering. This technology involves analyzing the structure, functions, and operation of a device, object, or system to discover its operating principles and then creating a representation of it in a different form, such as a digital model [1,16,18]. A key feature of reverse engineering is the use of physical objects, such as anatomical specimens or virtual data (e.g., CT scans), as the basis for design and manufacturing processes.

In the present study, this workflow was used to generate three patient-specific stabilizers adapted to the reduced C1–C2 anatomy: a clinical ventral C1–C3 design, a shortened ventral C1–C2 construct, and a clinical dorsal C1–C2 stabilizer. Each construct began with high-resolution CT segmentation of the cranial section of the cervical spine, followed by virtual reduction in the C1–C2 subluxation to obtain accurate alignment for implant fitting. This step was essential because anatomical alignment strongly influences screw angulation, plate curvature, and implant–bone contact zones. Once the vertebrae were aligned, their surfaces were converted into triangular mesh models, which served as the geometric foundation for plate design.

For all constructs, angular adjustments to the screws were necessary to optimize the available bone stock and minimize the risk of penetrating the vertebral canal. These adaptations were based on studies that identified safe corridors for the atlantoaxial junction, determining the optimal insertion points and angular variations to ensure proper inclination. Patient-specific screw trajectories were therefore created not only for biomechanical reasons but also to maintain adherence to known anatomical safety margins for both ventral and dorsal approaches. In the ventral stabilizers, particular attention was devoted to the orientation of screws inserted into the body of the axis, due to the narrow bone corridor and the proximity of neurovascular structures. For the dorsal construct, the screw vectors were tailored to the dorsal arch of C1 and the spinous process of C2 to ensure bicortical purchase without risking canal breach.

Following the design stage, 3D printing played a crucial role in evaluating prototypes. Anatomical models of C1–C3 were fabricated using Digital Light Processing (DLP) resin printing to verify implant fit, plate curvature, and screw alignment before metal production. These models enabled a hands-on assessment of the implant geometry, ensuring that plates followed the patient’s bone surface precisely and that the screws seated correctly within their planned safe corridors.

After validation, the implants were manufactured using metal additive manufacturing, specifically Selective Laser Melting (SLM), which allowed for the production of fully dense Ti-6Al-4V stabilizers with high geometric fidelity. The additive process enabled the creation of complex plate geometries with integrated features, such as curvature transitions and variable thicknesses, which would be challenging or impossible to achieve with traditional subtractive manufacturing. Post-processing steps, including support removal, locking holes, CNC machining, surface finishing, and cleaning, ensured that the final stabilizers met the mechanical and biocompatibility requirements expected in veterinary cervical fixation devices.

By integrating reverse engineering, patient-specific modeling, and additive manufacturing, this study demonstrates a modern workflow for producing bespoke atlantoaxial stabilizers. This process enabled rapid generation of multiple implant variants derived from the same anatomical case, allowing a rigorous comparative evaluation in the finite element environment while maintaining clinically realistic geometry.

### 4.3. Cadaver Study

A cadaver study of small and toy breeds revealed that dorsal instrumentation can be performed faster. Before printing the stabilizers, the screw insertion points were defined based on data obtained from CT scanning to optimize utilization of the bone stock and reduce the risk of penetrating the vertebral canal. This approach ensured that drilling remained confined within the bone stock, thereby avoiding fractures and/or implant failure.

Surgical or operative duration is an independent and potentially modifiable risk factor for complications. For instance, studies of various surgical procedures in humans have reported a positive correlation between procedure duration and complications, such as surgical site infections (SSIs), venous thromboembolisms, bleeding, hematoma formation, and necrosis [17]. Similarly, significant correlations have been found between the duration of surgical procedures in dogs and the incidence of postoperative complications. One of the most important of these is SSI. For instance, Cheng et al. [18] discovered that the likelihood of an SSI was twice as high for a 90 min surgical procedure compared to a 60 min procedure. Furthermore, Eugster et al. [19] found that the risk of an SSI doubled for every additional 70 min of surgery. Stetter et al. [20] also identified these correlations and recommended that surgical procedures should be as short as possible.

Dorsal instrumentation of the C1–C2 junction is less common but has been shown to yield good clinical outcomes [6,21]. Additionally, Cabreira et al. [9] demonstrated the feasibility of computationally developing a patient-specific implant designed for dorsal atlantoaxial fixation. Furthermore, the implant did not exhibit mechanical failure during FEA.

The cadaver study has some limitations. The time required for ventral or dorsal stabilization of the C1–C2 junction was measured using thawed fresh-frozen cadavers. Due to the time required to develop the stabilizer after CT scanning, we were unable to use fresh cadavers for technical reasons. In this instance, fresh-frozen cadavers were the only choice, as they are traditionally used for training in surgical procedures, offering lifelike anatomical conditions and realistic tissue handling. However, the freezing and thawing processes compromise the integrity of soft tissues. Bell et al. [22] found that human knee and shoulder tissue specimens from cadavers could be refrozen and used up to three times each without significant degradation of the critical tissues needed for arthroscopic simulation. However, notable changes were observed in the surrounding muscle and subcutaneous tissue when examined by ultrasound. In our study, the thawed cadavers were used only once. The use of live animals for experimental and educational purposes has decreased significantly in response to public concerns, meaning cadavers remain a common resource for practical training. Although formaldehyde-preserved cadavers are suitable for learning anatomy, they lack the characteristics necessary for surgical training on live patients, particularly for procedures requiring good tissue flexibility [23]. In this study, the flexibility of the cadavers’ soft tissues permitted an adequate surgical approach, enabling the use of drill guides and stabilizers at the C1–C2 junction. Compared to live animals, the absence of bleeding may have impacted the time taken to perform the procedure. Due to the need for hemostasis in both the ventral and dorsal approaches, operating times were likely longer. However, due to the nature of this study, the differences in bleeding intensity between the ventral and dorsal approaches are unknown. Another obvious limitation of the cadaver study is that it used dogs with no pathology at the C1–C2 junction. In most cases of AAI, ventral subluxation of the a Atlas is observed [6]. Before instrumentation can be performed, the subluxated vertebra must be reduced first. For ventral instrumentation, one method is to position the patient in dorsal recumbency and use padded support to elevate the cervical region. The thoracic limbs should then be placed caudally and secured to either side of the thorax. Finally, secure the upper jaw to the operating table with tape. Another commonly used intraoperative method involves inserting a screw into the body of the axis. After twisting the suture around the screw, the C2 vertebra can be lifted and retracted [14]. Retraction should continue until stable instrumentation of the C1–C2 junction is achieved. Another method of reducing C1–C2 subluxation involves using a Gelpi retractor fixed between the occipital bone and the C2–C3 intervertebral space as a reduction tool [16]. This can create more space for instrumentation, preventing collision with the lifting screw described above. To reduce C1 subluxation before dorsal instrumentation, it can be useful to position the dog in ventral recumbency with a pad under the neck and jaw to elevate the cervical vertebral column and head [20]. With both approaches, it can be assumed that adjusting the vertebral subluxation earlier would have prolonged the time required for the stabilizer to be applied. However, the nature of this study means that it is unclear whether the difficulty of reducing C1 subluxation and the time needed to do so differ between ventral and dorsal approaches. It appears that custom-made stabilizers can be used as reduction tools in a manner similar to precontoured or anatomical bone plates; however, this requires a clinical study in patients with atlantoaxial instability and subluxation of the Atlas [24,25].

This study found that dorsal instrumentation could be installed more quickly than ventral instrumentation, with comparable mechanical performance. However, the simulation does not model cyclic loading or fatigue limits. Also, cancellous bone was not modeled explicitly because its volume fraction is relatively small compared to the cortical shell of the C1 and C2 vertebrae in toy breeds [9,26]. For this reason, the available veterinary literature does not provide sufficient information to enable an extended load simulation to be carried out. It was also not possible to promote arthrodesis of the C1–C2 junction when the spinal stabilizer was inserted dorsally, which could be a significant clinical issue. Consequently, the long-term effectiveness of this construct may be limited. Therefore, clinical studies are necessary to verify whether C1–C2 arthrodesis is required to maintain the long-term stability of the atlantoaxial junction with the proposed dorsal stabilizer design. A final limitation of the cadaver study is the relatively small number of dogs operated on, which resulted in insignificant correlations between the time required to insert the stabilizer and the dog’s body weight, despite a clear tendency towards positive association. Anyway, it is likely that larger dogs require more time for instrumentation of the atlantoaxial junction. This finding, however, requires further research involving a larger group of dogs.

## 5. Conclusions

The combined findings from the numerical and cadaveric components of this study provide a comprehensive view of the biomechanical and practical considerations involved in atlantoaxial stabilization in toy-breed and small-breed dogs. By evaluating three patient-specific constructs—an extended ventral C1–C3 plate, a shortened ventral C1–C2 variant, and a dorsal C1–C2 stabilizer—the study demonstrates that all fixation strategies provide adequate stability within physiological load limits, while differing substantially in their internal load-transfer characteristics. The FEA showed that ventral constructs, particularly the C1–C3 configuration, distribute mechanical stresses more uniformly along the cervical column, reducing cortical loading of the atlantoaxial interface. The dorsal construct, in contrast, minimizes implant stresses but increases the mechanical demand on C1 and C2, reflecting a bone-centric load pathway.

Cadaveric instrumentation confirmed that all constructs can be implanted reliably with the aid of patient-specific guides, although anatomical accessibility and operative complexity vary between approaches. Together, these findings indicate that ventral stabilization may offer biomechanical advantages in cases requiring optimal long-term load distribution, whereas dorsal stabilization remains a viable and efficient alternative when ventral access is limited or when higher implant loading is undesirable. Future investigations, incorporating cyclic loading, mechanical testing of instrumented cadavers, and clinical follow-up, will be essential to validate these computational predictions and support the evidence-based selection of the most suitable stabilization strategy for individual patients.

## Figures and Tables

**Table 1:** Average mesh quality parameters of ventral and dorsal constructs.

Metric	Dominant Value in the Ventral Construct		Dominant Value in the Dorsal Construct	Interpretation
	C1–C3	C1–C2		
Element quality	0.82	0.82	0.80	High element quality
Aspect ratio	1.90	1.91	2.01	Acceptable aspect ratio range
Skewness	0.25	0.25	0.29	Low skewness (minimal distortion)

**Table 2:** Material properties used in the finite element analysis.

	Young’s Modulus [GPa]	Poisson’s Ratio
Ti-6Al-4V	110	0.3
Cortical Bone	12.54	0.3

**Table 3:** Contact definitions and friction coefficients used in the finite element models.

Interface	Contact Type	Friction Coefficient
Bone–bone	Frictional	0.46
Bone–plate	Frictional	0.30
Bone–screw	Bonded	-
Plate–screw	Bonded	-

**Table 4:** Finite element analysis results.

		The Ventral Construct		The Dorsal Construct
		C1–C3	C1–C2	
**5 N**				
**Stabilizer**	Displacement [mm]	0.08	0.01	0.02
	Strain [mm/mm]	0.002	0.0002	0.00005
	Stress [MPa]	58.83	11.44	4.70
**C1**	Displacement [mm]	0.01	0.01	0.03
	Strain [mm/mm]	0.0001	0.0005	0.0008
	Stress [MPa]	1.38	4.46	6.05
**C2**	Displacement [mm]	0.08	0.01	0.02
	Strain [mm/mm]	0.003	0.0009	0.002
	Stress [MPa]	16.51	8.12	15.02
**C3**	Displacement [mm]	0.01	-	-
	Strain [mm/mm]	0.002	-	-
	Stress [MPa]	12.33	-	-
**10 N**				
**Stabilizer**	Displacement [mm]	0.15	0.01	0.05
	Strain [mm/mm]	0.003	0.0004	0.0001
	Stress [MPa]	114.67	21.4	9.89
**C1**	Displacement [mm]	0.20	0.02	0.06
	Strain [mm/mm]	0.0002	0.001	0.001
	Stress [MPa]	2.76	8.48	13.59
**C2**	Displacement [mm]	0.16	0.01	0.03
	Strain [mm/mm]	0.005	0.002	0.003
	Stress [MPa]	32.83	14.76	30.48
**C3**	Displacement [mm]	0.02	-	-
	Strain [mm/mm]	0.004	-	-
	Stress [MPa]	24.83	-	-
**15 N**				
**Stabilizer**	Displacement [mm]	0.23	0.02	0.07
	Strain [mm/mm]	0.005	0.0007	0.0002
	Stress [MPa]	166.17	49.03	14.57
**C1**	Displacement [mm]	0.30	0.03	0.08
	Strain [mm/mm]	0.0003	0.002	0.002
	Stress [MPa]	4.03	20.14	24.12
**C2**	Displacement [mm]	0.30	0.01	0.05
	Strain [mm/mm]	0.009	0.003	0.005
	Stress [MPa]	57.14	20.28	46.32
**C3**	Displacement [mm]	0.03	-	-
	Strain [mm/mm]	0.005	-	-
	Stress [MPa]	32.51	-	-
**20 N**				
**Stabilizer**	Displacement [mm]	0.30	0.03	0.10
	Strain [mm/mm]	0.006	0.001	0.002
	Stress [MPa]	211.83	49.21	22.41
**C1**	Displacement [mm]	0.40	0.04	0.11
	Strain [mm/mm]	0.0005	0.002	0.003
	Stress [MPa]	5.51	21.86	31.71
**C2**	Displacement [mm]	0.31	0.02	0.07
	Strain [mm/mm]	0.01	0.004	0.01
	Stress [MPa]	84.33	20.12	60.58
**C3**	Displacement [mm]	0.03	-	-
	Strain [mm/mm]	0.007	-	-
	Stress [MPa]	50.33	-	-
**25 N**				
**Stabilizer**	Displacement [mm]	0.38	0.04	0.12
	Strain [mm/mm]	0.007	0.001	0.0003
	Stress [MPa]	255.51	62.21	24.29
**C1**	Displacement [mm]	0.49	0.05	0.14
	Strain [mm/mm]	0.0007	0.003	0.004
	Stress [MPa]	7.26	25.8	38.32
**C2**	Displacement [mm]	0.39	0.02	0.09
	Strain [mm/mm]	0.02	0.005	0.007
	Stress [MPa]	116.68	26.78	77.63
**C3**	Displacement [mm]	0.04	-	-
	Strain [mm/mm]	0.009	-	-
	Stress [MPa]	62.52	-	-

## Data Availability

The original contributions presented in this study are included in the article/Supplementary Materials. Further inquiries can be directed to the corresponding authors.

## Supplementary Materials

The following supporting information can be downloaded at https://www.mdpi.com/article/10.3390/ma19020316/s1. Figure S1: Ventral C1–C3 construct, Figure S2: Ventral C1–C2 construct, Figure S3: Dorsal construct, Figure S4: Results of finite element analysis.

## Abbreviations

The following abbreviations are used in this manuscript:

FEA	Finite Element Analysis
SLM	Selective Laser Melting
