# Supplementary figures and images for: A Comparison of Two Surgical Treatment Methods for Atlantoaxial Instability in Dogs: Finite Element Analysis and a Canine Cadaver Study
Source: Materials (Basel). 2026 Jan 13;19(2):316. doi: 10.3390/ma19020316 (PMC12843234; doi:10.3390/ma19020316)

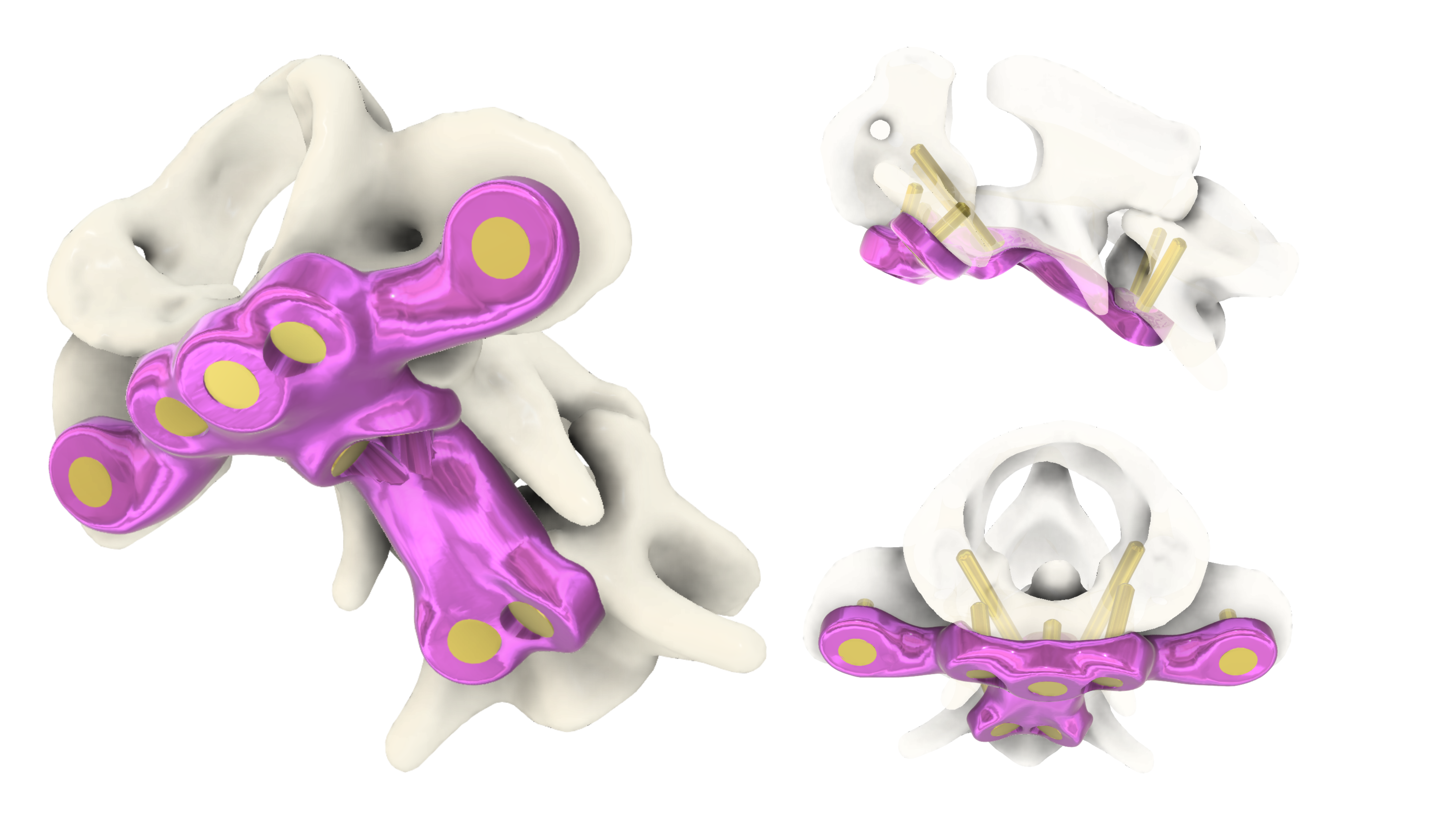

Supplement: Supplementary file 1 [file materials-19-00316-s001.zip › Figure S1 Ventral C1-C3 construct.png]

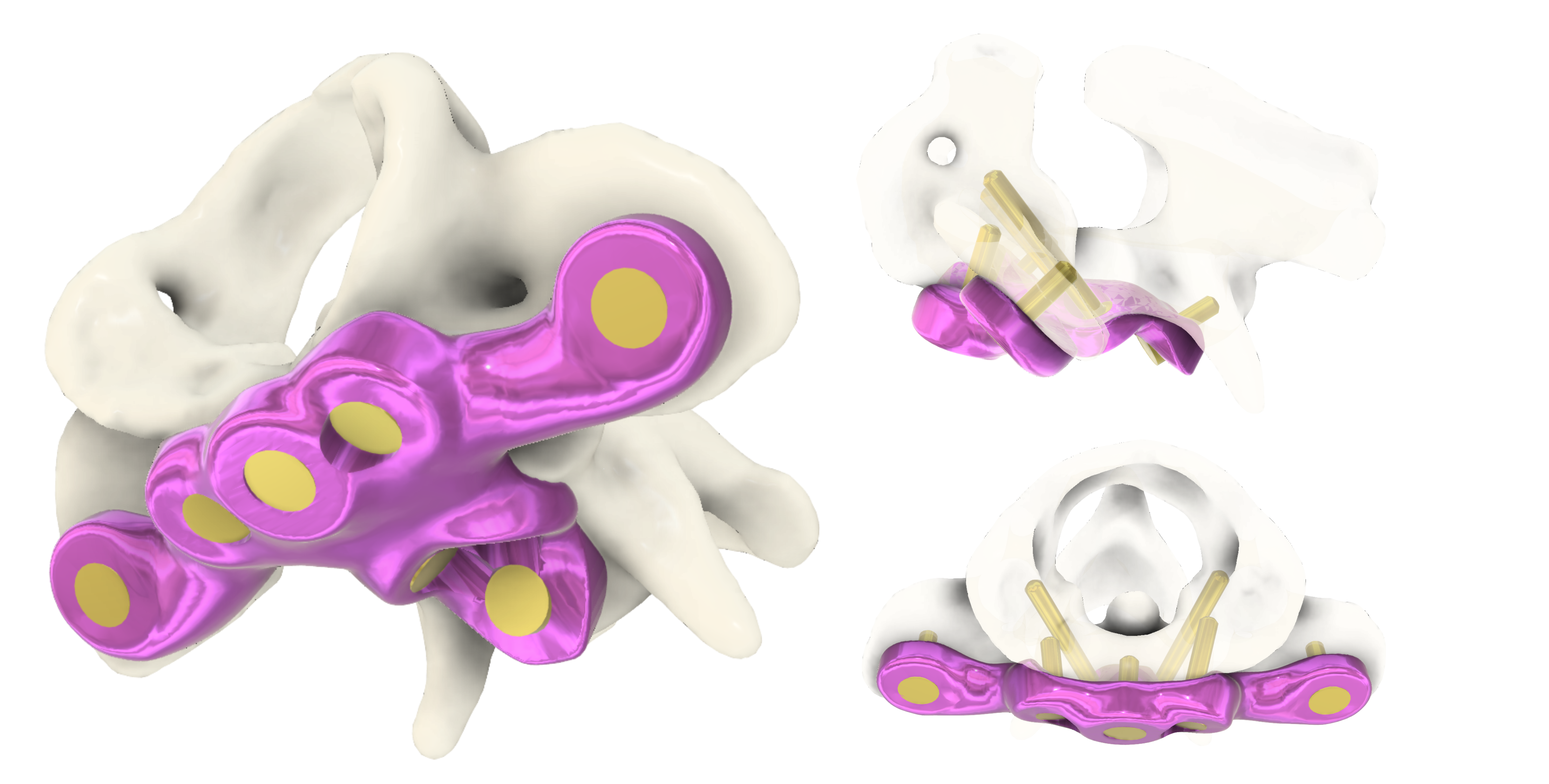

Supplement: Supplementary file 1 [file materials-19-00316-s001.zip › Figure S2 Ventral C1-C2 construct.png]

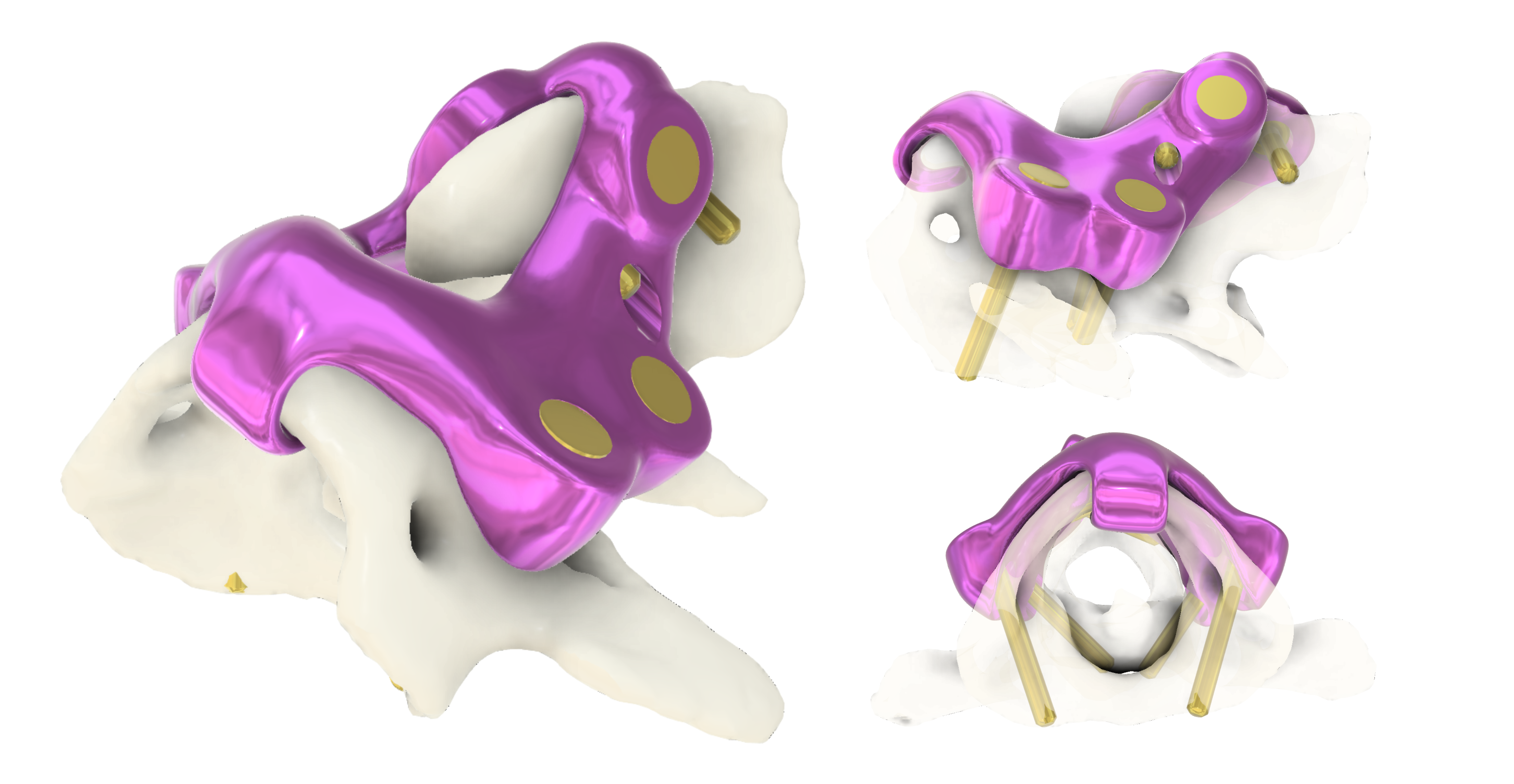

Supplement: Supplementary file 1 [file materials-19-00316-s001.zip › Figure S3 Dorsal construct.png]
